# Restoration Strategies of Endodontically Treated Teeth among Dental Practitioners in Saudi Arabia. A Nationwide Pilot Survey

**DOI:** 10.3390/dj6030044

**Published:** 2018-09-03

**Authors:** Abdulrahman Alenzi, Abdulaziz Samran, Ahlam Samran, Mohammad Zakaria Nassani, Mustafa Naseem, Zohaib Khurshid, Mutlu Özcan

**Affiliations:** 1 Private Clinic, Riyadh 13314, Saudi Arabia; thary32@hotmail.com; 2 Department of Restorative and Prosthetic Dental Sciences, College of Dentistry, Dar Al Uloom University, Riyadh 13314, Saudi Arabia; asamran@dau.edu.sa (A.S.); aasamran@yahoo.com (A.S.); 3 Department of Prosthetic Dental Sciences, Al Farabi College for Dentistry and Nursing, Riyadh 13224, Saudi Arabia; mznassani@hotmail.com; 4 Department of Removable Prosthodontics, Faculty of Dentistry, University of Aleppo, Aleppo 15310, Syria; 5 Department of Preventive Dental Sciences, College of Dentistry, Dar Al Uloom University, Riyadh 13314, Saudi Arabia; mustafanaseem86@gmail.com; 6 Prosthodontics and Dental Implantology, College of Dentistry, King Faisal University, Al Ahsa 31982, Saudi Arabia; 7 Dental Materials Unit, Center for Dental and Oral Medicine, Clinic for Fixed and Removable Prosthodontics and Dental Materials Science, University of Zürich, 8032 Zürich, Switzerland; mutluozcan@hotmail.com

**Keywords:** endodontically-treated teeth, post type, dental practitioners, restoration

## Abstract

The purpose of this study was to determine dental practitioners’ opinions, techniques, and materials used for the restoration of endodontically treated teeth (ETT) in Saudi Arabia. A comprehensive nationwide survey regarding treatment strategies of ETT, on the post types and material used for core foundations were distributed either by email or by hard copies to general dentists in different parts of Saudi Arabia (North, South, West, East, and Center). Descriptive statistics were used to analyze the responses to the questions. A total of 164 participants were included in the survey: 72.6% of them were male, and 27.4% were female. 42.1% of the participants were Saudi dental practitioners, whereas 57.9% were non-Saudi dental practitioners. Out of the surveyed dentists, 52% consider post placement for almost every post-endodontic restoration of ETT. The majority of the dentists (54%) believe that a post strengthens ETT. Cast posts and cores were used by 55% of all the dentists, whereas 34% used prefabricated posts exclusively. Screw posts were the most popular prefabricated post type (47%). Composite resin (51%) was preferred for the core foundation, followed by glass ionomer cements (GICs) (26%). Amalgam was seldom used (0.5%). Posts were placed primarily with zinc phosphate cement (51%), followed by GIC (38%). Within the limitations of this survey-based investigation among dental practitioners in Saudi Arabia, it was concluded that the treatment strategies of ETT are in accordance with the current state of evidence-based knowledge.

## 1. Introduction

Endodontic treatment is carried out for teeth affected by caries, repeated restorations, and fractures. A definite restorative solution is necessary after this type of therapy. Substance loss in endodontically treated teeth (ETT), due to caries or the endodontic procedure, increases the susceptibility of the tooth to fracture [1]. Therefore, the longevity of such teeth is dependent on the amount of substance loss and the ability of the restorative materials to replace the missing tooth structure [2]. Furthermore, multiple parameters may also affect the prognosis of ETT, i.e. the remaining tooth structure [3,4], the type of final restoration, the design of the post and core material used [5,6], and the presence of a ferrule [3,7].

Different strategies have been proposed for the restoration of ETT [4,8,9,10]. These treatment strategies include the use of posts and cores [4], partial or full coverage crowns [11], and direct resin composites or amalgam fillings [12]. Several post systems are available for the restoration of ETT. The use of fiber posts is popular because they have a similar modulus of elasticity to that of dentin [13]. In addition, they have good physical properties and can be removed easily [14].

Posts are recommended when the coronal structure is insufficient to support a core build-up [4]. A study by Sorensen and Martinoff showed that full coverage crowns did not significantly improve the clinical success for anterior teeth, whereas they improved the success rate for premolars and molars [15]. The substantial loss of ETT structures during tooth preparation worsens the situation when endodontic treatment is associated with mesioocclusodistal (MOD) cavities. At this point, adhesive restorations, which have the potential to reinforce weakened tooth structure, seem to have an advantage over crowns [16].

Several survey-based studies have been published on the strategies and preferences of dental practitioners in terms of restoring ETT. Such data exist for the United Kingdom [17], Sweden [18], the United States [19], Switzerland [20], India [21], Panevezys [22], and Germany [23]. In addition, there are several materials and techniques advocated for restoring ETT. However, the strategies and preferences of dental practitioners in Saudi Arabia in terms of restoring ETT need to be investigated. Therefore, the aim of this nationwide survey-based investigation was to assess the strategies and preferences for restoring ETT among dental practitioners in Saudi Arabia.

## 2. Materials and Methods

### 2.1. Involvement of the Participants

The questionnaire used in the current study was modified from a similar study performed in Germany in 2006 [23]. The questionnaire survey was mailed (as a soft copy) and given out by hand (as hard copies) to 300 dental practitioners anonymously throughout Saudi Arabia. The dentists who received hard copies were visited within a period of 3 months (January–March 2017) by the first author (AA), who described the objective of the survey to the participants. Each participant was then given the questionnaire and information leaflet on the study procedures. The dental practitioners were asked to return the completed surveys by mail (for the soft copy) or by hand (for the hard copies). The participating dental practitioners were selected randomly within five regions in Saudi Arabia (North, South, West, East, and Center). The participants did not receive any further compensation and the non-responders were not reminded due to the anonymous character of the questionnaire. Out of a total of 300 participants, 164 dental practitioners returned the completed questionnaire, resulting in a response rate of 54.67%.

### 2.2. Questionnaire

The first part of the questionnaire related to basic demographic details. The second part of the questionnaire contained 14 multiple-choice questions focused on the treatment strategies of ETT and the materials and methods used for the treatment. The questionnaire consisted of the following questions:

**1. Do you use posts in the treatment of ETT?** - Yes - No
**2. Which type of posts do you usually use?** - Prefabricated posts - Custom-made cast posts and cores - I do not use posts in the treatment of my patients.
**3. Do you believe that every endodontically treated tooth (ETT) must receive a post?** - Yes - No - I do not know.
**4. Do you believe that a post reinforces an ETT and reduces the fracture probability?** - Yes - No - I do not know.
**5. Do you believe that creating a ferrule below the core foundation following post cementation increases fracture resistance?** - Yes - No - I do not know.
**6. In your opinion, what is the main criterion in choosing between prefabricated posts and custom-made posts?** - The remaining tooth structure - Ease of use - A reduced number of visits - Ease of removal when a problem has occurred - Cost - Aesthetic purposes - Tooth location (anterior or posterior) - Canal width - Other.
**7. If you use prefabricated posts, which type do you mostly use?** - Fiber-reinforced composite posts (FRC) (glass fiber posts, carbon fiber, mixed) - Metal-based pots (screws, titanium posts, stainless steel) - Ceramic-based posts (zirconia posts) - I do not use any type of the above - Others.
**8. When you use a prefabricated metal post, which design do you mostly prefer?** - Parallel-sided - Tapered - Combined parallel-sided/tapered - Screw type - Split flexible posts - Threaded - I do not use prefabricated metal posts - Other.
**9. Which type of custom-made post and core do you mostly use?** - Gold cast – Base-metal alloy -Titanium - Zirconia - I do not use custom-made cast posts - Other.
**10. What type of cement do you use for post cementation?** - Dual polymerized adhesive resin cement - Chemically polymerized adhesive resin cement - Self-adhesive resin cement - Glass ionomer - Resin-modified glass ionomer cement - Zinc phosphate cement - Polycarboxylate cement - Other.
**11. What type of core build-up material do you mostly prefer to use with prefabricated posts?** -Resin composite - Glass ionomer build-up material - Amalgam - Other.
**12. When do you usually insert the posts into the canal after obturation?** - Directly 24 h after obturation - One week post obturation - After several weeks post obturation - Other.
**13. Should the tooth (with post and core) be crowned?** - Yes - No - I do not know.
**14. Which crown material do you usually indicate?** - Full metallic crown - Full ceramics – Porcelain-fused-to-metal (PFM) - Other.

### 2.3. Statistics

The data collected were analyzed using descriptive statistics (SPSS 22.0 for Windows; SPSS, Inc., Chicago, IL, USA). Frequency distributions (for treatment strategies including standard deviation) were used for the descriptive statistical representation of the results.

## 3. Results

### Demographic and General Information

**Figure 1:**
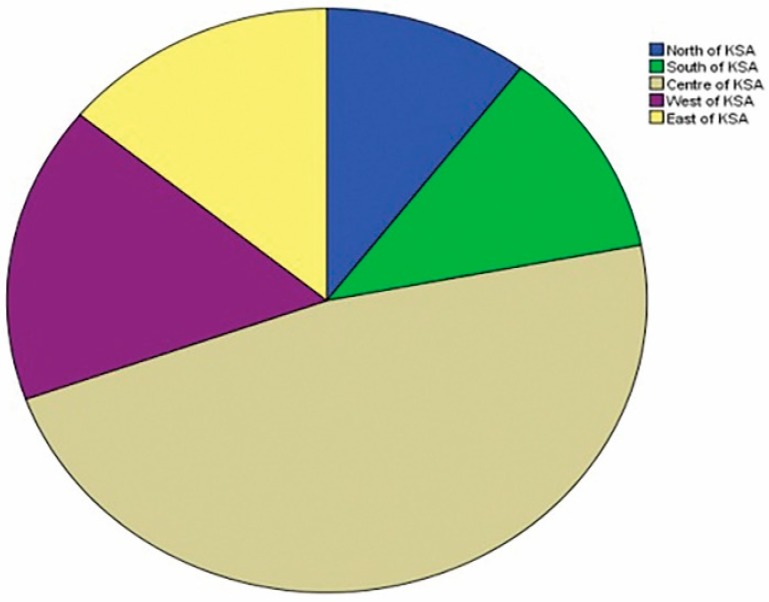
Percentage values of the participants according to practice location.

A total of 164 questionnaires regarding the treatment strategies for the restoration of ETT were completed in five main regions in Saudi Arabia as follows: North 10.4%; South 11.6%; Center 47.5%; West 16.5%; and East 14% (Figure 1). In total, 109 (66.5%) of the responders were general practitioners, whereas 55 (33.5%) were specialists or consultants. A total of 119 (72.6%) of the responders were men, while 45 (27.4%) were women. The responders’ ages ranged between 25 and 63, and their working experience ranged between 1 and 35 years. Out of the responders, 95 (57.9%) were non-Saudi dentists, whereas 69 (42.1%) were Saudi dentists.

**1.**  **Frequency of Use of Dental Posts in ETT**

A total of 161 (98.2%) of the responders utilized posts in the treatment of ETT; however, 3 (1.8%) of them did not use posts with ETT.

**2.**  **Type of Posts**

According to the responses, 138 (84.1%) of the responders utilized prefabricated posts in the treatment of ETT, and 26 (15.9%) of them utilized custom-made cast posts and core ETT.

**3.**  **Belief That Every ETT Must Receive a Post**

Regarding treatment concepts, 149 (90.9%) of the dentists did not believe that all ETT must receive a post. However, 13 of the dentists (7.9%) believed that every ETT must receive a post and 2 (1.2%) did not know.

**4.**  **Belief That Posts Strengthen ETT**

**Figure 2:**
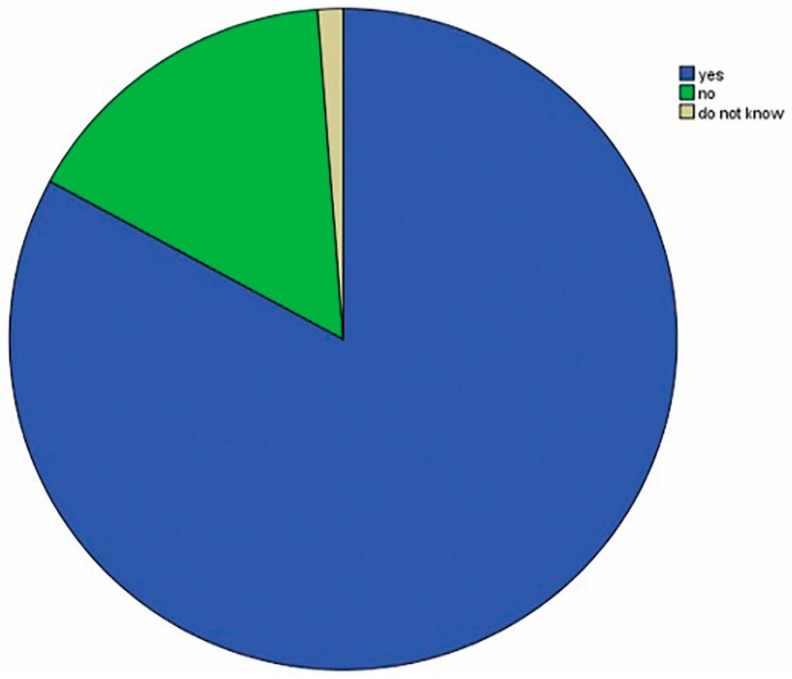
Percentage values of the participants according to whether the post reinforces ETT or not.

Surprisingly, 136 (82.9%) of the responders believed that the presence of a post would strengthen ETT and therefore decrease the risk of fracture. However, 26 (15.9%) of the responders did not believe that a post would improve the fracture strength of ETT and 2 (1.2%) did not know (Figure 2).

**5.**  **The Importance of a Ferrule below the Core Foundation**

**Figure 3:**
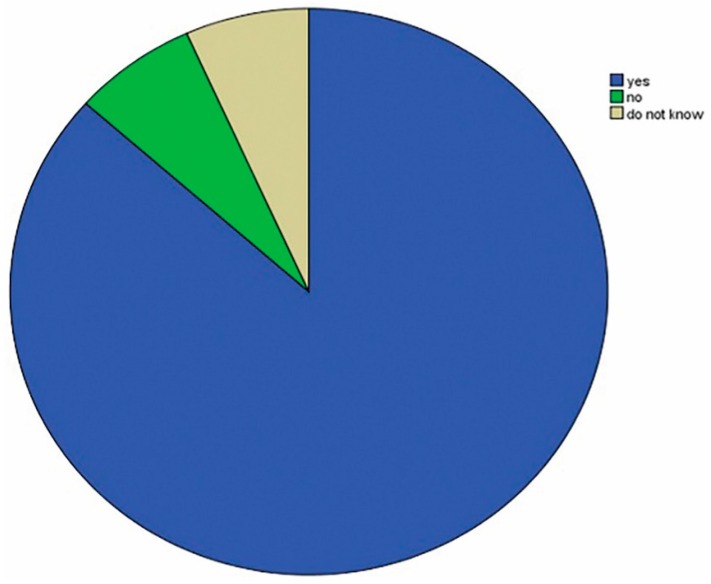
Percentage values of the participants according to whether the ferrule below the core foundation following post cementation will increase the fracture resistance of ETT or not.

Approximately, 142 (86.6%) of the participants believed that the presence of a ferrule below the core foundation following post cementation would increase the fracture resistance of ETT. However, 11 (6.7%) of them did not believe that it would improve the fracture resistance of ETT and 11 (6.7%) did not know (Figure 3).

**6.**  **The Main Criterion in Choosing between Fiber Posts and Custom-Made Posts? (Which Decision Criteria Led to the Preferred Use of Fiber Posts or Custom-Made Posts and Core System?)**

Based on the respondents’ answers, the main criteria that led to the preferred use of fiber posts or custom-made posts and core systems were: The remaining tooth structure (77.4%); ease of use (7.3%); fewer visits (3.7%); cost (1.8%); aesthetic purposes (0.6%); tooth location (anterior or posterior) (2.4%); canal width (2.4%); and others (4.3%).

**7.**  **Type of Prefabricated Posts**

**Figure 4:**
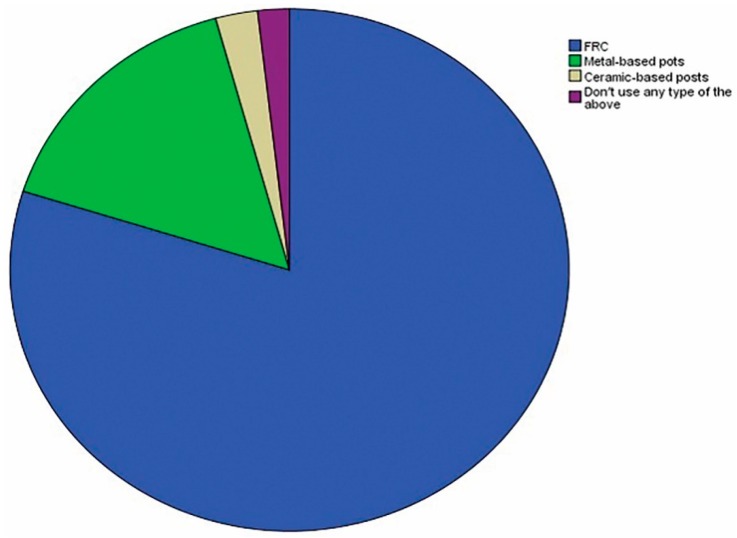
Percentage values of the participants according to the type of prefabricated posts used.

Regarding prefabricated post types, 131 (79.9%) of the respondents preferred fiber-reinforced composite posts (FRC), 26 (15.9%) opted for metal-based posts (such as titanium or stainless steel), and 4 (2.4%) favored ceramic-based posts. Around 3 (1.8%) of the respondents stated that they did not use any prefabricated posts (Figure 4).

**8.**  **Design of Prefabricated Metal Posts**

Concerning the design of metal prefabricated posts, 98 (59.8%) of the respondents preferred a tapered design, 17 (10.4%) preferred a parallel-sided design, 15 (9.1%) chose a screw design, 5 (3%) opted for a combined parallel-sided/tapered design, 2 (1.2%) favored a threaded design, and 1 (0.6%) preferred a split flexible design. Interestingly, 26 (15.9%) of the respondents stated that they did not use prefabricated metal posts.

**9.**  **Type of Custom-Made Post and Core**

Regarding the type of custom-made post and core, 103 (62.8%) of the respondents utilized a base-metal custom-made cast post and core. Around 10 (6.1%) of the dentists utilized a titanium custom-made cast post and core, 7 (4.3%) of the respondents utilized a gold custom-made cast post and core, and 6 (3.7%) utilized a zirconia CAD/CAM custom-made post and core. However, 38 (23.2%) of the respondents stated that they did not use a custom-made post and core.

**10.**  **Type of Cement Used for Post Cementation**

In terms of the type of cement used, 56 (34.1%) of the respondents preferred dual polymerized adhesive resin cement, 56 (34.1%) opted for self-adhesive resin cement, 21 (12.8%) favored chemically polymerized adhesive resin cement, 16 (9.8%) preferred glass ionomer cement, 14 (8.5%) preferred resin-modified glass ionomer cement, and 1 (0.6%) preferred zinc phosphate cement.

**11.**  **Type of Core Build-Up Material Most Commonly Used with Prefabricated Posts**

Regarding the type of core build-up material most commonly used with prefabricated posts, 153 (93.3%) of the respondents preferred resin-based core materials, 9 (5.5%) preferred glass ionomer materials, 1 (0.6%) preferred amalgam, and 1 (0.6%) preferred another material.

**12.**  **Time of Post Insertion**

The majority of the respondents (72%) stated that they preferred to prepare the canal for the post during the first week after obturation. However, 22.6% of the respondents reported that they preferred to prepare the post space directly within 24 h after obturation. The rest of the respondents (i.e., 3%) stated that they preferred to prepare the post space after several weeks of obturation.

**13.**  **Should the Tooth (with Post and Core) Be Crowned?**

The majority of the surveyed practitioners (95.1%) stated that they crown ETT after treatment with a post and core. Around (4.3%) of the participants would not place a crown on ETT after using a post and core. However, only 0.6% of the surveyed practitioners answered that they did not know.

**14.**  **Crown Material Used over ETT with Post and Core**

Approximately 53% of the surveyed participants would place a full ceramic crown as the restoration of choice for ETT with a post and core. However, 46.3% preferred to place a porcelain-fused-to-metal (PFM) crown for ETT with a post and core, and only 0.7% stated that they would place a full metal crown.

## 4. Discussion

This nationwide pilot survey was conducted to update the recent treatment strategies and materials used in ETT restoration in Saudi Arabia. Since the survey was kept anonymous, it was not possible to remind practitioners who did not return the questionnaire. However, the participation rate in the study was 54.67%. The response rate was found to be in concurrence with other studies using the same methodology [19,23].

According to the results of this survey-based investigation, the majority of the participants (98.2%) used an endodontic post during the treatment of ETT. In addition, this study found that the greater part of the participants (84.1%) preferred to use prefabricated posts over custom-made posts and cores, which corresponds with the observations of other similar studies [24,25]. This trend may be due to the fact that prefabricated posts are easy to use and can be completed in a single visit. Amongst prefabricated posts, the FRC post was used by the majority of the dentists. This could be due to the fact that fiber posts have a similar modulus of elasticity to that of dentin, which may decrease the chances of root fracture in ETT [3,26,27]. The present investigation revealed that the majority of the surveyed dental practitioners (90.9%) were not in agreement with the opinion that all ETT should be treated with a post and core restoration, which is in line with the consensus in literature [23,28,29]. This result can be attributed to the fact that teeth with less substance loss do not need posts to retain the final restoration [4].

Astoundingly, the present study validated that 82.9% of the participants believed that the presence of a post could strengthen ETT and therefore decreases the risk of fracture. This finding was found to be in harmony with the dental practitioners practicing in Sweden, Germany, the United States, and India [18,19,21,23]. However, it is at odds with current evidence-based investigations, which reveal that posts do not reinforce ETT [4,30]. The percentage of the respondents who believed in the reinforcement effect of ferrule averaged approximately (86.6%), which was found to be in concurrence with scientific literature, where the presence of ferrule is considered as a cornerstone for avoiding clinical failures [2,6,31,32,33].

There are a number of factors that influence the choice of post type (prefabricated or custom-made cast post and core), i.e., the remaining tooth structure, ease of use, a reduced number of visits, cost, aesthetic purposes, and tooth location. Most of the participants in the present survey reported that the main decision criterion leading to the preferred use of a fiber post or custom-made post and core system was the remaining tooth structure (77.4%). One conceivable explanation for this finding is that the greater the tooth substance loss, the greater the need for custom-made posts and cores, which facilitates close adaptation to the post space more than prefabricated posts. Unfortunately, in current literature, no clear recommendations exist regarding this question.

Regarding the prefabricated post type, 79.9% of the respondents in this investigation preferred using prefabricated FRC posts over metal-based posts (15.9%). This result may be attributed to the fact that fiber posts have a similar modulus of elasticity to that of dentin, can distribute stresses more evenly along the post-dentine interface, and tend to cause reparable root fractures when they fail [14,22,31]. Non-rigid posts, on the other hand, have a high modulus of elasticity like metals and can produce stress concentration at critical areas of the root which may lead to irreparable root fractures [31,32,33]. In contrast, dental practitioners in Germany, Sweden, and Switzerland preferred prefabricated metal posts [18,20,23].

Concerning the design of metal prefabricated posts, the majority of the respondents (59.8%) in the present survey favored a tapered design, whereas 10.4% of the respondents preferred a parallel-sided design, and only 9.1% chose a screw design. This finding may be accredited to the fact that special burs are needed for the parallel-sided preparation of the canals. In addition, screw designs are not preferred due to their disadvantages as the higher incidence of root fractures lowers the survival rate significantly [34,35]. Dental practitioners in Germany and Switzerland, on the other hand, preferred the screw type [20,23]. However, practicing dentists in Sweden opt for parallel-sided prefabricated metal posts [18].

Regarding the type of custom-made post and core, 62.8% of the participants utilized a base-metal custom-made cast post and core. This result can be attributed to the cheaper cost of the material compared with gold and titanium alloys or custom-made zirconia posts and cores. In the present investigation among dental practitioners, the most common cement used to lute the posts was resin-based cement (68.2%). In a comparable study among dental practitioners in Switzerland [20], the use of zinc phosphate cement was found to be more popular. However, the majority of dental practitioners in the United States and Northern Ireland preferred glass ionomer cement [17,19]. The result of the present study can be explained by the fact that resin-based materials exhibited more favorable physical and chemical properties than glass ionomer cements and zinc phosphate cements [26,36].

Regarding the type of core build-up material most commonly used with prefabricated posts, the majority of the participants (93.3%) preferred resin-based materials followed by glass ionomer core materials (5.5%). This result was in agreement with the results of a survey-based investigation in Germany and the United States [19,23]. However, it was not in line with the results of a comparable study carried out in the United Kingdom, where amalgam core material was more popular [17]. This may be due to the fact that amalgam materials are still not considered as a health threat to the patient and have a good level of acceptability. In addition, resin-based composite core materials are more popular due to their chemical bond to the tooth structure and similarity to the tooth structure in terms of hardness and fracture toughness, offering the advantage that preparation can be performed after curing [37].

It was apparent that the majority of the participants (72%) usually prepare the canal for the post during the first week after obturation. However, 22.6% of the respondents reported that they preferred to prepare the post space directly within 24 h after obturation. This is at odds with the current state of evidence-based knowledge, which reports that the time elapsed between canal obturation and post cementation significantly influences fiber post retention [38].

The majority of the surveyed practitioners (95.1%) stated that they crown ETT after treatment with a post and core. This finding can be explained by the fact that ETT are more prone to fracture than vital teeth due to the removal of tooth structure during endodontic treatment and fractured or carious dentin, meaning that they need to be crowned to save the integrity of the structure.

Concerning crown materials, more than half (53%) of the respondents would opt to place a full ceramic crown as the restoration of choice for ETT with a post and core. However, 46.3% of them reported that they would place a PFM crown for ETT with a post and core, and 0.7% stated that they would place a full metal crown. The shift among the dentists toward all-ceramic restorations in the present study can be explained by the need to meet the increased demands of patients and dentists for highly aesthetic and biocompatible restorations [39].

A limitation of this survey-based study is that it did not distinguish between the restoration of anterior ETT and posterior ETT. A future study with a larger sample size is highly recommended to confirm the findings of this pilot survey.

## 5. Conclusions

Within the limitations of this study, the following conclusions can be drawn:

- Most of the participants in the present study did not consider that ETT should receive a post. However, most of them believed that ETT should be crowned, and all-ceramic crowns were the preferred choice.
- The majority of the surveyed dentists stated that the presence of a post could reinforce ETT.
- The decision to utilize any type of post was mainly based on the remaining tooth structure.
- Most of the participants believed that the presence of a ferrule would increase the fracture resistance of ETT.
